# Supplementary material for: Direct observation of altermagnetic band splitting in CrSb thin films
Source: Nat Commun. 2024 Mar 8;15:2116. doi: 10.1038/s41467-024-46476-5 (PMC10923844; doi:10.1038/s41467-024-46476-5)
Supplement: Supplementary file 1 — Supplementary Information [file 41467_2024_46476_MOESM1_ESM.pdf]

SUPPLEMENTARY INFORMATION: Direct observation of altermagnetic band splitting in CrSb thin films

S. Reimers,[1] L. Odenbreit,[1] L. Šmejkal,[1] V. N. Strocov,[2] P. Constantinou,[2] A. B. Hellenes,[1] R. Jaeschke Ubiergo,[1] W. H. Campos,[1] V. K. Bharadwaj,[1] A. Chakraborty,[1] T. Denneulin,[3] Wen Shi,[3] R. E. Dunin-Borkowski,[3] S. Das,[4,5] M. Kläui,[1,6] J. Sinova,[1,7] and M. Jourdan*[1]

[1]*Institut für Physik, Johannes Gutenberg-Universität Mainz, 55099 Mainz, Germany*
[2]*Paul Scherrer Institut, CH-5232 Villigen PSI, Switzerland*
[3]*Ernst Ruska-Centre for Microscopy and Spectroscopy with Electrons, Forschungszentrum Jülich, 52425 Jülich, Germany*
[4]*Department of Physics and Astronomy, George Mason University, Fairfax, VA 22030, USA*
[5]*Center for Quantum Science and Engineering, George Mason University, Fairfax, VA 22030, USA*
[6]*Centre for Quantum Spintronics, Norwegian University of Science and Technology NTNU, 7491 Trondheim, Norway*
[7]*Department of Physics, Texas A&M University, College Station, Texas 77843-4242, USA*

SUPPLEMENTARY INFORMATION

Characterization of the epitaxial CrSb(100) thin films

X-ray diffraction (XRD) analysis of epitaxial CrSb thin films in the Bragg-Brentano geometry, as depicted in Fig. S1, unequivocally confirms their (100)-orientation and exceptional phase purity. The CrSb (h00)-peaks are the only visible X-ray peaks besides the peaks originating from the GaAs substrate and sample holder. All observed peaks, both in specular (panel **a**) as well as in off-specular geometry (panel **b**) agree well with PowderCell XRD simulations assuming a hexagonal CrSb crystal structure with space group no. 194 and lattice parameters $a = 4.1$Å and $c = 5.58$Å, as shown in Fig. 1. The XRD rocking curve shown in panel **c** indicates a moderate mosaicity (tilt range of the 001-axis) of $\simeq 0.5^o$ resulting in broadening of the ARPES data in $k_{\|}$. It is worth noting that, as per the results obtained from the PowderCell simulations, the scattering intensities of observable peaks in the specular geometry represent only a minute fraction in comparison to the strongest off-specular peak. Specifically relating to the strongest off-specular XRD-peak, which is the (101) peak, the simulation yields the following relative intensities of the specular peaks shown in Fig. S1: (100): 0.2%, (200): 0.08%, (300): 7.49%.

The in-plane orientation and the existence of a highly structured atomic arrangement at the sample surface are exemplified through the inclusion of the reflection high-energy electron diffraction (RHEED) image, inserted as an inset within Fig. S1.

The high degree of crystallographic ordering in the bulk of the thin film samples is further underscored by the scanning transmission electron microscope (STEM) images, presented in Fig. S2.

Panel **a** provides a perspective along the [001] in-plane direction, panel **b** along the [100] in-plane direction of a CrSb(100) epitaxial thin film, with the CrSb lattice depicted for reference. Notably, there are no discernible signs of disorder. It is important to acknowledge that

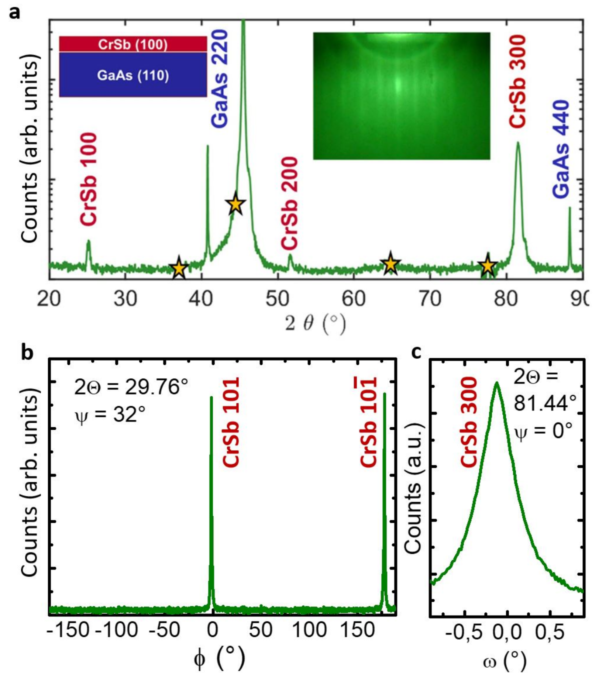

Supplementary Figure 1. **X-ray and electron diffraction. a** $\Theta/2\Theta$ scan of a 30 nm CrSb(100) epitaxial thin film grown on a GaAs(110) substrate. The inset shows the RHEED image of the sam-ple, demonstrating a well-ordered sample surface with full in-plane orientation. **b** ϕ-scan of the off-specular CrSb 101 and $10\bar{1}$ peaks showing the in-plane order of the sample with the associated 2-fold in-plane symmetry. **c** Rocking curve of the specular CrSb 300 peak showing a moderate mosaicity of$\simeq 0.5^o$

.

due to the inherent thickness averaging within the TEM lamella, the interchangeability of the local Sb environment within the antiferromagnetic Cr sublattices remains beyond visualization. Thus, we cannot make any statement about the size of sample regions with identical local Sb environment of the Cr sublattices.

Some degree of disorder at the CrSb(100) surface (interface with the SiN capping layer), specifically the ab-

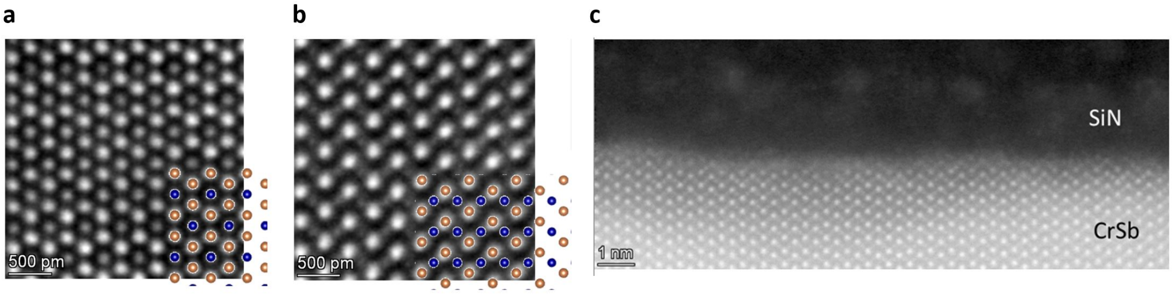

Supplementary Figure 2. **STEM images of an epitaxial CrSb(100) thin film.** **a**: view along [001]-direction, **b**: view along [100]-direction, with corresponding superimposed representation of the crystal structure (blue: Cr atoms, orange: Sb atoms.) **c**: view along [100]-direction in the surface/ upper interface region probed by SX-ARPES.

sence of a well-defined termination, is visible in panel **c**. Regarding the photoemission results, the associated limited translational invariance parallel to the sample surface is presumably the origin of blurring observed in the experimental results. Furthermore, the reduced surface order is likely to quench potential surface states.

Evaluating intensity line profiles though columns of atoms oriented perpendicular to the CrSb/Si_3N_4 interface, we identified a reduced distance (5-15%) between the last two Sb layers at the interface compared to the deeper layers. Such lattice distortions in the surface region could explain the discrepancies remaining in Fig. 4d (of the main manuscript) between the band structure calculations and the ARPES results.

For obtaining the STEM images, an electron transparent cross-section lamella was prepared using a 30 kV focused Ga+ ion beam and scanning electron microscope (FIB-SEM) FEI Helios platform. The ion beam energy was decreased to 5 kV for the final thinning steps. Scanning transmission electron microscopy (STEM) was carried out using a TFS Spectra TEM equipped with a Schottky field emission gun operated at 300 kV, a CEOS probe aberration corrector and a high angle annular dark-field detector (HAADF).

Identification of the bands along P-Q-P

The altermagnetically split bands appear only faint in the raw data. However, they are directly visible in the symmetrized ARPES intensity (raw data) shown in Fig. S3**a**. Panel **c** shows line profiles of the intensity at k-values indicated by the yellow lines in panel **a**. The upper branch of the altermagnetically split band shows up as a shoulder feature in these profiles. Consistent with the altermagnetic band structure, this shoulder feature is absent for the profiles obtained at the Q and P points. A side-by-side comparison of panels **a** and **b** confirms that the application of the Laplace filter emphasizes the bands correctly. The “spots” in the filtered image correspond to crossing points of bands, which result in an increased emission intensity.

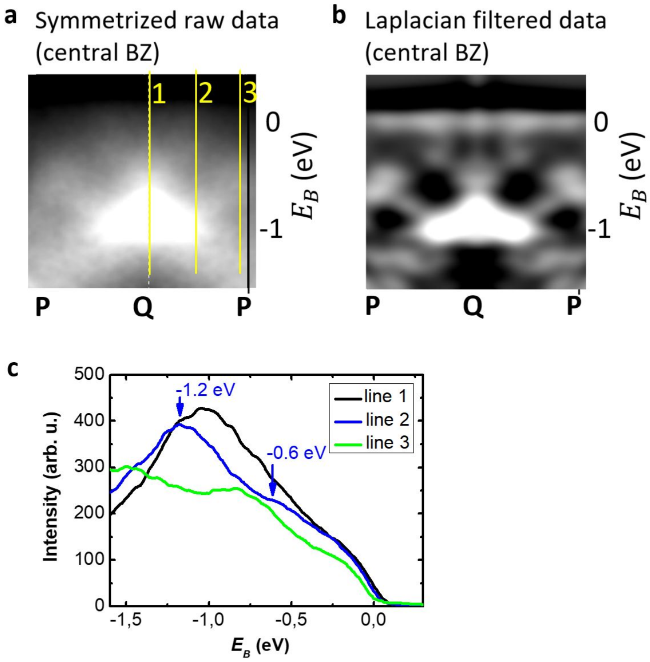

Supplementary Figure 3. **Identification of bands in the raw data.** **a**: Symmetrized ARPES raw data $I(E, \mathbf{k})$ along P-Q-P with the three yellow lines indicating the k-positions at which line pro-files of I were evaluated. Panel **b** shows the corresponding Lapace filtered data for direct comparison. **c**: Line profiles of the ARPES intensity (raw data) from panel **a**, with the intensity maximum of line 2 corresponding to the minimum of the lower branch of the altermagnetically split band and the shoulder feature corresponding to the upper branch.

One reason for the observed band broadening in the experimental data is the mosaicity of the epitaxial thin films shown in Fig. S1**c**, with a rocking curve width of$\simeq 0.5^{\circ}$ corresponding to $\Delta k_{\|} \simeq 0.1$Å for 900 eV photons. Another possible contribution to broadening effects both in energy as well as in momentum could be a relatively small size of sample regions with fully single crystalline order, specifically including the coherent local order of the Sb environment of the Cr sublattices. As swapping the local environment swaps the CEF at the Cr sublat-

tices, this is expected to specifically show up in the characteristic altermagnetic band structure features.

This in general to be expected effect also result in a swapping of the spin polarization of the altermagnetically split bands, even within a single magnetic domain. Thus, it presents a major challenge for potential future spin-resolved ARPES investigations, as averaging over such growth domains will result in averaging the spin polarization to zero.

Orbital character of the valence states

To identify the orbital character of the electronic bands showing the altermagnetic splitting, we show in Fig. S4**a** the projections on the Cr as well as on the Sb atomic orbitals. The dominant Cr character is obvious from the comparison of the calculated spectral weights of the orbitals. This result is fully consistent with the experimentally observed periodicity of the ARPES intensity from BZ to BZ, as discussed in the main text. Furthermore, we show in panel **b** the projections of the electronic bands on the s-, p-, and d-orbitals of Cr. We observe, that the major contribution originates from d-orbitals, which we discuss below in the framework of selection rules in photoemission spectroscopy.

Selection rules in photoemission along along $\mathbf{Q-P}$ in CrSb(100)

Selection rules governing photon polarization are applicable when the scattering plane in the experimental setup aligns with a mirror plane inherent to the crystal structure under investigation [31]. In Fig. S5**a**, the geometry of our photoemission experiment is shown. Both vector of grazing photon incidence indicated by the green line and the k-dispersive direction of the energy analyzer define the scattering plane shown in red. For the given sample orientation required for probing the Q-P path, this scattering plane coincides with a mirror plane of CrSb as shown in Fig. S5b (Cr atoms shown in blue, Sb in orange). Thus, for p-polarization of the photons, the photoemission intensity is dominated by electronic states, which are even with respect to the scattering/ mirror plane. An example of such a state is the $d_{x^2-y^2}$ orbital shown in the schematic representation of our set-up.

We have observed the characteristic altermagnetic band structure features along the P-Q-P path with p-polarised, but not with s-polarised photons. To check the consistency of this experimental result with the band structure calculations, we now discuss the spectral weight of the projections of the CrSb electronic states on both the atomic Cr and Sb orbitals. Our calculations clearly show that the spectral weight of the valence band is dominated by the Cr states. Thus, we plot the projection on these in Fig. S5.

Panel **d** shows the projections on the Cr d-orbitals, which are even with respect to the scattering/ mirror plane indicated in panels **a** and **b**, i. e. on those, which are selected by the p-polarised photons in the ARPES experiment. Panel **e** shows those, which are odd, i. e. which are selected by s-polarised photons. Comparing the projections with the full band structure calculation superimposed to the experimental data, it is obvious that the characteristic altermagnetically split band has mainly the character of the d-orbitals, which are even with respect to the scattering/ mirror plane of the experiment. In particular, the strongest contribution stems from the $d_{x^2-y^2}$-orbital indicated in panel **a**. Thus, the experimental result and the band structure calculations are fully consistent.

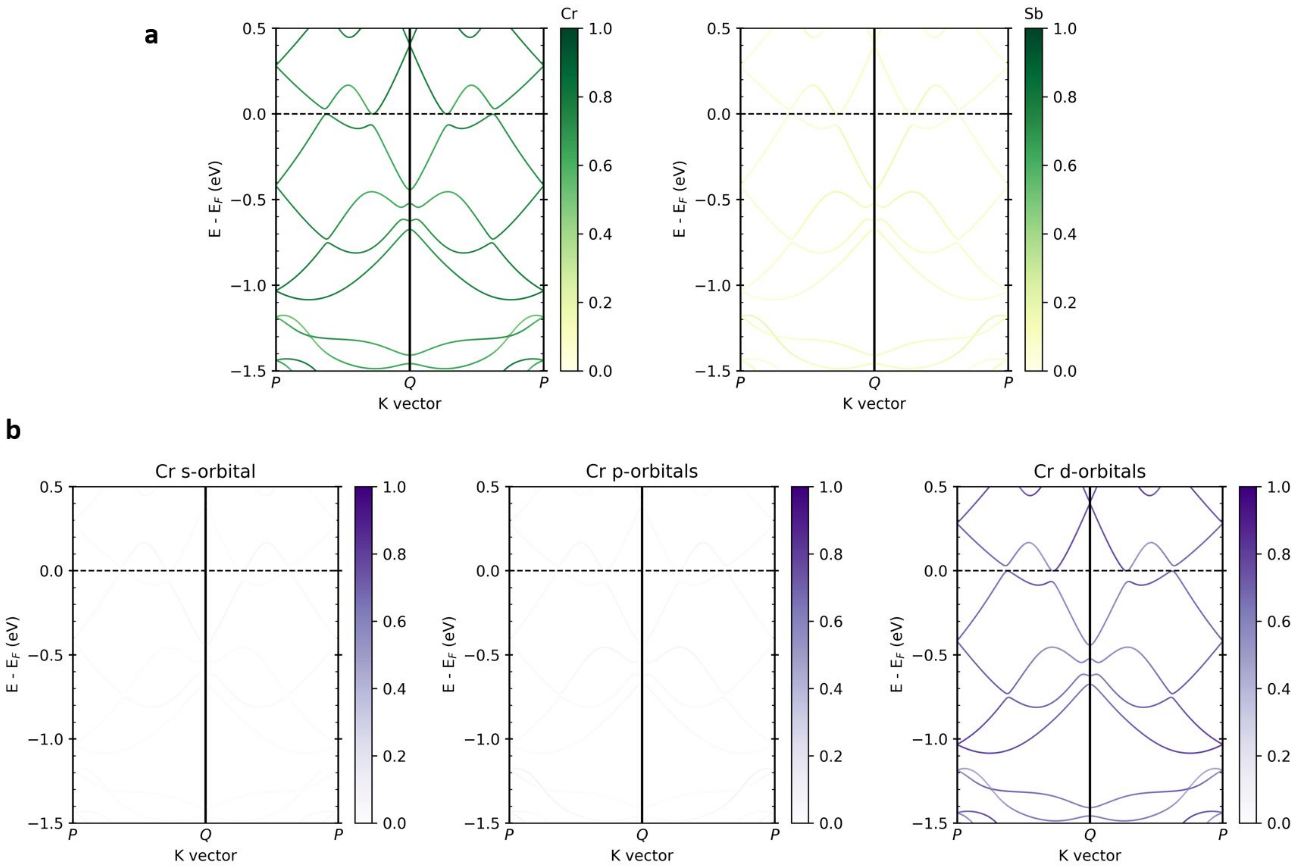

Supplementary Figure 4. **Orbital character of the electronic states. a**: Projection of the electronic states on the Cr (left) and Sb (right) atomic orbitals. The color scale indicates the spectral weight. **b** Projection of the Cr s- (left), p- (center) and d-orbitals (right). The color scale indicates the spectral weight, i. e. bands with low spectral weight appear faint in the plots

.

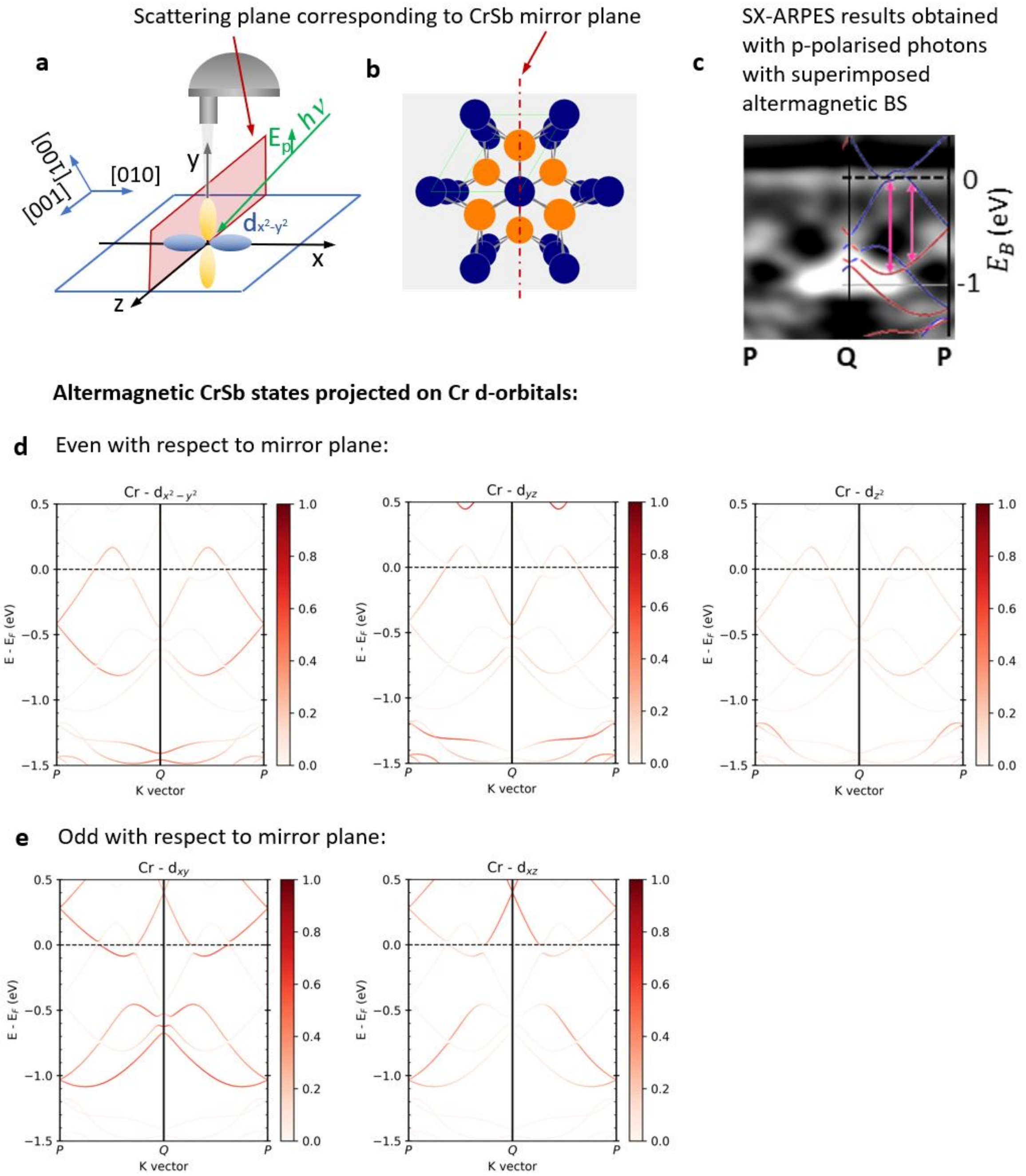

Supplementary Figure 5. **Experimental geometry and selection rules.** **a**: Schematic representation of the experimental geometry. The scattering plane (y,z-plane) contains both the incoming photon beam indicated by the green arrow (p-polarisation, i. e. po-larisation parallel to the scattering plane) as well as the angular dispersive direction of the detector. The blue plane shows the sample surface (x,z-plane) and the blue arrows indicate the orientation of the CrSb lattice, i. e. , the CrSb(100) thin film is oriented with its [001]-direction parallel to the z-axis as indicated. **b**: Perspective representation of a CrSb unit cell, view parallel to the [001]-axis (blue: Cr atoms, orange: Sb atoms). **c**: SX-ARPES results obtained with p-polarised photons and superimposed altermagnetic band structure. **d**: Projection of the CrSb electronic states on Cr d-orbitals, which are even with respect to the scattering/ mirror plane. These are selected by the p-polarisation of the photon beam. The color scale indicates the spectral weight. **e**: Projection of the CrSb electronic states on Cr d-orbitals, which are odd with respect to the scattering/mirror plane (selected by s-polarised photons).
